# The effects of physical therapy with neuromuscular electrical stimulation in patients with septic shock: Study protocol for a randomized cross-over design

**DOI:** 10.1097/MD.0000000000009736

**Published:** 2018-02-09

**Authors:** Alessandra Fabiane Lago, Anamaria Siriani de Oliveira, Hugo Celso Dutra de Souza, João Santana da Silva, Anibal Basile-Filho, Ada Clarice Gastaldi

**Affiliations:** a Department of Physiotherapy, Rehabilitation and Functional Performance Post Graduation Program; b Department of Biochemistry and Immunology; c Division of Intensive Care Medicine, Department of Surgery and Anatomy, Ribeirao Preto Medical School, University of Sao Paulo, Ribeirao Preto, Brazil.

**Keywords:** electric stimulation therapy, endothelial progenitor cells, indirect calorimetry, physical therapy modalities, septic shock

## Abstract

**Introduction:**

Septic shock is a potentially fatal organ dysfunction caused by an imbalance of the host response to infection. The changes in microcirculation during sepsis can be explained by the alterations in the endothelial barrier function. Endothelial progenitor cells (EPCs) are a potential recovery index of endothelial function and it an increase in response to neuromuscular electrical stimulation (NMES) was demonstrated. Therefore, the objective of this study is to investigate the effects of NMES in patients with septic shock.

**Methods and analysis:**

It is a study protocol for a randomized cross-over design in an intensive care unit of a tertiary University hospital. Thirty-one patients aged 18 to 65 years. The study will be divided in 2 phases: the phase one will be held in the first 72 hours of septic shock and the phase two after 3 days of first assessment. Patients will be randomly selected to the intervention protocol (decubitus position with the limbs raised and NMES) and control protocol (decubitus position with the limbs raised without NMES). After this procedure, the patients will be allocated in group 1 (intervention and control protocol) or group 2 (control and intervention protocol) with a wash-out period of 4 to 6 hours between them. The main outcome is mobilization of EPCs. The secondary outcome is metabolic and hemodynamic data. A linear mixed model will be used for analysis of dependent variables and estimated values of the mean of the differences of each effect.

## 1 Introduction

Septic shock is a potentially fatal organ dysfunction caused by an imbalance of the host response to infection. The incidence of septic shock is 18.6 cases per 1000 hospital admissions with a mortality rate of 50.7% in the United States of America.^[1]^ According to international guidelines, patients with septic shock can be identified with clinical signs of sepsis and persistent hypotension requiring a vasopressor to maintain mean arterial pressure ≥ 65 mm Hg and serum lactate levels >2 mmol/L (18 mg/dL) despite adequate fluid resuscitation.^[2–4]^

Changes occur during sepsis in microcirculation, and consequently, there is a decreased blood supply resulting in organ dysfunction. This fact can be explained by the alterations in endothelial barrier function during disease.^[5]^ Indeed, the endothelium is responsible for the interface between tissues and the blood compartment and participates in the local regulation of blood flow and vascular caliber.^[6]^ Studies on the endothelial function and endothelial progenitor cells (EPCs) have been carried out in different groups of patients with metabolic syndrome, cardiovascular disease, in hemodialysis patients as well as in healthy individuals.^[7–9]^ The EPCs are a specific subtype of hematopoietic stem cells that can migrate from the bone marrow into the peripheral circulation. These cells are a potential recovery index of endothelial function and play an important role in neovascularization, repair, and restoration of the endothelium.^[8]^

In addition, it is common to use sedation and mechanical ventilation in septic shock patients. The frequent use of sedation limits mobilization of critically ill patients usually showing a progressive loss of muscle strength and functionality. Physiotherapy can use different techniques to help these patients increase muscle strength, recovery of functional status, decrease the length of weaning of mechanical ventilation, and decrease the duration of delirium.^[10,11]^ One of these techniques used to recruit muscles in the intensive care environment is neuromuscular electrical stimulation (NMES). The NMES is directed to those patients who cannot cooperate with physiotherapy due to sedation or decreased muscle strength, and it allows the patients to perform exercises in passive, assisted, and active modes.^[12]^ This noninvasive therapy is applied regardless of the patient's effort and produces a muscle contraction in response to a externally generated low-voltage electric impulse, which is conducted through electrodes placed on the skin over the muscle group to be stimulated.^[12,13]^

Some studies have shown evidence that the electrical stimulation reduces muscle atrophy and the time of weaning from mechanical ventilation and therefore increases muscle strength in critically ill people^[14–16]^; beneficial results of NMES have been demonstrated in patients with sepsis. In these patients, Rodriguez et al^[17]^ showed improved muscle strength—especially those in critical condition. Stefanou et al^[18]^ demonstrated an increase in EPCs after NMES. In patients with septic shock, only 1 study has evaluated the effects of NMES^[19]^; it demonstrated a marked decrease in the quadriceps volume independently of the use of NMES. However, the benefits of NMES are not limited only to the maintenance of muscle strength.^[14,15]^ Literature findings suggest that there is a potential benefit of NMES in acute activation of microcirculation observed by the increased rate of oxygen consumption and reperfusion as measured by infrared spectroscopy.^[20,21]^

Combining the pathophysiology of septic shock, the role of EPCs, the effects of NMES, and the divergence of results of the studies using NMES in patients in septic shock, we hypothesized that NMES initiated an early phase of septic shock and could contribute favorably to the mobilization of EPCs without causing metabolic and hemodynamic impairments. Therefore, the objective of this study is to investigate the effects of NMES in patients with septic shock.

## 2 Patients and methods

### 2.1 Study design

This study is a randomized controlled crossover clinical trial with group 1 (starts with intervention and ends with control protocol) and group 2 (control and intervention protocol).

### 2.2 Study participants and eligibility criteria

The patients admitted to the intensive care unit are eligible if they present in the first 72 hours after the diagnosis of septic shock according to the international consensus definition of septic shock. They should have stable hemodynamic through fluid resuscitation, vasoactive drugs, and mechanical ventilation as described above.

Exclusion criteria will include patients aged 18 years or less or over 65 years, pregnant women, brain death, neuromuscular diseases, or the use of a pre-existing neuromuscular blocker in the last 24 hours.

Contraindications for the use of NMES will include fractures, burns, skin lesions, systemic vascular impairment diseases such as systemic lupus erythematosus, thromboembolic disease, deep vein thrombosis (which was not therapeutically anticoagulated for more than 36 hours), lower limb amputations, cardiac pacemaker, thrombocytopenia less than 20,000/mm^3^, body mass index greater than 35 kg/m^2^, important lower extremity edema, agitation, and/or signs of pain during the electrical stimulation.

Contraindications to begin or continue NMES procedure will include the following: mean arterial blood pressure less than 65 mm Hg, use of vasopressor >50% of the maximum dose (dopamine >12.5 μg/kg per minute; vasopressin >0.02 U/min and norepinephrine >1 μg/kg per minute), heart rate <50 or >140 bpm, arrhythmias with hemodynamic consequences, myocardial ischemia, temperature <34^ o^C or >39^o^C, intracranial pressure >20 cmH_2_O, decrease in 10% peripheral oxygen saturation (SpO_2_) baseline value or <88% for more than 1 minute.

### 2.3 Recruitment organization

All patients with a diagnosis of septic shock will be recruited at intensive care unit admission. An explanatory statement will be given and writing informed consent will be obtained before the commencement of the study. The author AFL will have final approval of a patient's eligibility for the study. If the relatives of the patients accept the invitation to participate in the study, then they will sign the informed consent form.

### 2.4 Randomization and allocation

This study will follow the recommendations of the Consolidate Standards of Reporting Trials (CONSORT)^[22]^ and the study protocol followed the Stardard Protocol Items for Randomised Trials (SPIRIT).^[23]^ After the consent, AFL will evaluate and determine the eligibility of patients. Thus, ACG will not be involved with the assessment and interventions that randomly allocates the participants in 1 of the 2 groups through a simple randomization (random numbers generated by the computer). The allocation sequence will be hidden by ABF through sequential numbered opaque sealed envelopes. After this step, AFL will open the envelope and will start the protocol. Blood sample analyses will be blinded to AFL, ACG, and ABF.

### 2.5 Procedures

**Figure 1:**
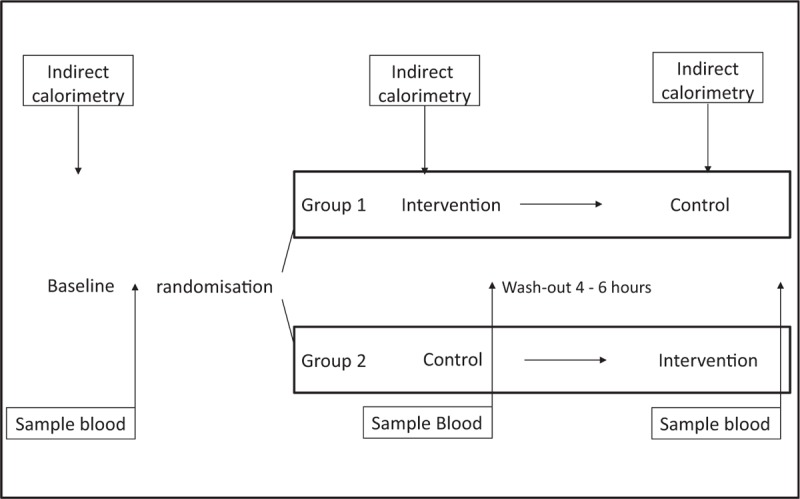
Protocol design.

The study will be divided into 2 phases, the phase one will be held in the first 72 hours of septic shock; the phase two will be 3 days after the first assessment. Patients will be submitted in a random order to the intervention protocol (decubitus position with the limbs raised and NMES) and control (decubitus position with the limbs raised without NMES). The patients may be allocated in Group 1 (intervention and control) or group 2 (control and intervention) with a wash-out period of 4 to 6 hours between them as visualized in Fig. 1.

The study has a short follow-up, and the second measurements will be done after 3 days. Patients who discontinue or deviate from protocol are in the second phase of study; only results of the phase one of the study will be analyzed. These results will demonstrate the acute effects of NMES in the first 72 hours after diagnosis of septic shock.

### 2.6 Primary outcomes

#### 2.6.1 Endothelial progenitor cells (EPCs)

To evaluate the EPCs, blood samples will be taken from an existing central venous access. The first 3 mL is discarded, and the remainder will be collected in 5 mL tubes with acid citrate and dextrose 5 minutes before the baseline and at the end of each protocol. The samples will be processed within 3 hours after collection and will be kept at 4°C during the procedure. The analysis will be performed by flow cytometry (BD Biosciences, FACS Canto ^TM^ II, EUA) and centrifuged at 700g for 20 minutes with no brake. The upper phase (plasma) will be removed gently and aliquoted at 0.25 mL. The lower phase containing the blood cells will be resuspended using 10 mL of cold 1 × PBS containing 0.5% (w/v) of BSA and 1.5 mM EDTA and it will be centrifuged again at 700g for 20 minutes with braking. The upper stage will be removed and discarded, the cell sediment will be resuspended, and 2.5 mL will be transferred to a separate tube and kept on ice.

Concurrently, 500 μL of sample will also be transferred in an isotype control and in 3 sample tubes with the appropriate antibodies. It will then be added to 9 mL of ACK lysis buffer to lyse red blood cells and incubated at room temperature (18°C–25°C) for 3 minutes. It will be washed twice with 9 mL cold 1 × PBS and finally centrifuged to 250*g* to 4°C with no brake for 5 minutes. Cell sediment will be resuspended twice in 500 μL of 1× PBS, and the samples will be filtered through 40 μm cell strainer in BD 5 mL Falcon tubes. Tubes will be kept at 4° C (or ice) in the dark before the flow cytometry. Different subpopulations of endothelial progenitor cells will be quantified by cellular markers by flow cytometry: CD34+/CD133+/CD45−, CD34+/CD133+/CD45−/VEDFR_2_e CD34^+^/CD45−/VEGFR_2_+.^[24,25]^ Nurses will receive a training in how blood sample will be taken, even as laboratory staff will be trained to evaluate cell progenitor endothelial.

### 2.7 Secondary outcomes

#### 2.7.1 Oxygen consumption (VO_2_), carbon dioxide production (VCO_2_), and resting energy expenditure (REE)

Indirect calorimetry (IC) is a noninvasive method that analyzes the amount of heat generated by the whole body according to the substrate utilization. The data provided by calorimetry are resting energy expenditure (REE) that is calculated from the amount of VO_2_ and VCO_2_ through the respiratory gases. The inspired fraction of oxygen is measured through the inspiratory branch of a mechanical ventilator. The expiratory gases pass through a mixing chamber where the fraction of expired air of oxygen and carbon dioxide are analyzed.^[26]^ Patients will be submitted to IC during baseline and study protocol. The IC will be measured by a portable calorimeter DELTATRAC II Metabolic Monitor (Datex-Ohmeda, Helsinki, Finland) connected to a mechanical ventilator (Evita XL, Dräger medical, Lübeck, Germany) for 30 minutes in a stable condition without manipulation of the upper airways or changes of the ventilator parameters. The intensive care unit staff will be trained in how to proceed when the IC is running. We consider steady state to be the point after 5 consecutive minutes measurement when oxygen consumption and carbon dioxide production vary by ±10%. This technique was employed in some of our previous studies^[27–29]^ and was validated elsewhere.^[30]^ The protocol will initiate after warming the calorimeter for 30 minutes. The gas and pressure (95% O_2_/5% CO_2_) is calibrated according to the manufacturer's instructions.

### 2.8 Other outcomes

#### 2.8.1 Hemodynamic variables

The change in hemodynamic and respiratory variables will be measured, including heart rate, blood pressure, oxygen saturation, and breathing frequency. The change in cirtometry will be collected by the measurements of the circumference of the gastrocnemius muscle.

#### 2.8.2 Interventions

The protocols will be carried out in adult intensive care center at a tertiary University Hospital.

#### 2.8.3 Intervention protocol – decubitus position with the limbs raised and NMES

**Figure 2:**
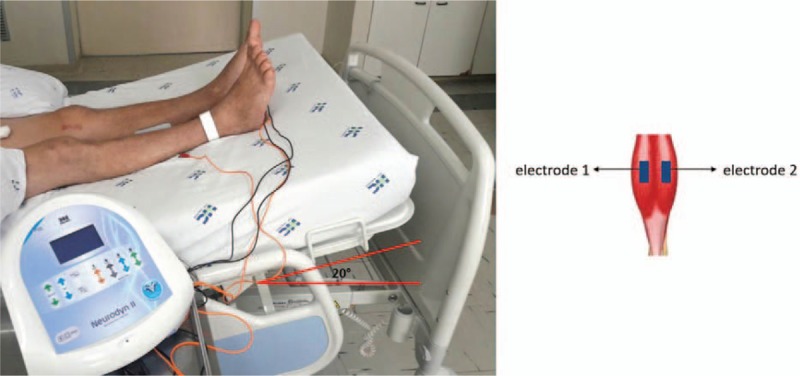
Intervention protocol.

The patient will be positioned on a headboard at 30° in the decubitus position with the limbs raised to 20°. The location of the electrical current will be cleared with trichotomy when necessary. Adhesive electrodes 90 x 50 mm will be positioned in the gastrocnemius. The stimulator device will be the Neurodyn II (Ibramed, Sao Paulo, Brazil) to provide symmetrical biphasic pulses of 50 Hz, 250 μsec pulse duration, 2 seconds on (1 second of time of rise and 1 second of time of decay), and 5 seconds of rest during 30 minutes at an intensity capable of generating visible contractions and articular motion (Fig. 2).

#### 2.8.4 Control protocol – decubitus position with the limbs raised without NMES

The patient position will be the same used for intervention protocol (headboard to 30°, decubitus position with the limbs raised to 20° for 30 minutes.

Adverse event will be defined as any change in mean arterial blood pressure less than 65 mm Hg, heart rate <50 or >140 bpm, arrhythmias with hemodynamic consequences, myocardial ischemia, decrease in 10% of SpO_2_ baseline value, or <88% for more than one minute and pain during the NMES session. Pain will be evaluated through the Brazilian version of the Behavioural Pain Rating Scale^[31]^ to measure pain in unconscious patients under mechanical ventilation and unable to communicate verbally. If any adverse event happen, the session will be interrupted and the event will be related.

This study is a trial with a short duration and with known minimal risks; therefore, data-monitoring committee is not necessary.

#### 2.8.5 Data analysis and management

All data will be entered electronically. There will be a subject identification code list and each participant will receive a participant study identification number. Confidential documents will be retained in the institution's computer in the laboratory. A password system will be utilized to control access. Back up of data will be kept in locked cabinets in the laboratory as well.

The data set will be described via tables and figures containing values expressed as mean, standard deviation, median, minimum, and maximum values. The mean effects of the protocol and the group differences for all outcomes will be calculated using linear mixed models to incorporate terms for the protocols, sequence, and periods; a term referring to the carryover effect will also be added in order to control these effects.^[32]^

The effects will be related to differences of values in relation to the protocols, the sequences and the periods and it will be measured considering the value from the first moment of protocol and the value in the second time. The effects are listed below:

- The Protocols: Protocol 1: referring to intervention. Protocol 2: regarding the control.
- Sequences/Groups: Sequence/group 1: Related the sequence at the beginning of the study with the decubitus position with the limbs raised and NMES and ending with decubitus position with the limbs raised without NMES (Group 1). Sequence/group 2: Related to the sequence at the beginning of the study with the decubitus position with the limbs raised without NMES and ending with the decubitus position with the limbs raised and NMES (Group 2).
- Periods: period 1: related to the time of the first procedure. Period 2: related to the moment of the second procedure.

Estimated values and 95% confidence intervals are used to evidence the possible effects related to the studied factors. The *P* value is used to identify whether or not there was an effect of the protocol, the sequence, and the period. The level of significance will be set at 5%. Statistical analysis will be performed through the R version 3 (2013-05-16) (The R Foundation for Statistical Computing, Vienna, Austria) and SAS version 9.2 (SAS Inc., Cary, NC).

The sample size of 31 patients was based on the study of Stefanou et al^[18]^ for the variable CD34 + CD133 +, CD45− with effective detection of 7.3/10^6^ cells, standard deviation of 10.2, 5% alpha, and power of 80%.

The investigators will provide verbal and written feedback to relatives of participating patients about the results of the study and clinical conditions.

Any modifications to the protocol and administrative changes of the protocol will be communicated to ClinicalTrials.gov and Research Ethics Committee and clinical research unit of the Hospital das Clínicas da Faculdade de Medicina de Ribeirão Preto.

All principal investigators will have access to the final trial dataset.

## 3 Discussion

Septic shock is a potentially fatal organ dysfunction caused by an imbalance of the host response to infection. This disease is life-threatening circulatory failure with inadequate tissue perfusion because changes may occur in microcirculation and occurs alterations in endothelial barrier function.^[5]^

Indeed, the endothelium is responsible for the interface between tissues and the blood compartment and participates in the local regulation of blood flow and vascular caliber.^[6]^ Studies have been shown the relation between EPCs and their neovascularization capability in adult ischemia tissue and participation in the formation of new blood vessels.^[7,33]^

Physiotherapy can use NMES in critically ill patients. It has been demonstrated that NMES is well tolerated and it seems to have a short-term systemic effect on the peripheral microcirculation, potential benefit acute activation of microcirculation observed by the increased rate of oxygen consumption and reperfusion as measured by infrared spectroscopy.^[20,21]^

Therefore, this work will demonstrate the possible beneficial effects of electrical stimulation as evaluated by the mobilization of endothelial progenitor cells in patients with septic shock. It will determine whether electrical stimulation can be applied in critically ill patients without causing harmful metabolic and/or hemodynamic changes.

## Acknowledgment

The authors thank laboratory and intensive care staff assistance during the preparation of this manuscript.

## References

[1] Kadri SS Rhee C Strich JR Estimating ten-year trends in septic shock incidence and mortality in United States Academic Medical Centers using clinical data. Chest 2017;151:278–85. 27452768 10.1016/j.chest.2016.07.010 PMC5310115

[2] Shankar-Hari M Phillips GS Levy ML Developing a new definition and assessing new clinical criteria for septic shock: for the Third International Consensus Definitions for Sepsis and Septic Shock (Sepsis-3). JAMA 2016;315:775–87. 26903336 10.1001/jama.2016.0289 PMC4910392

[3] Singer M Deutschman CS Seymour CW The Third International Consensus Definitions for Sepsis and Septic Shock (Sepsis-3). JAMA 2016;315:801–10. 26903338 10.1001/jama.2016.0287 PMC4968574

[4] Seymour CW Liu VX Iwashyna TJ Assessment of clinical criteria for sepsis: for the Third International Consensus Definitions for Sepsis and Septic Shock (Sepsis-3). JAMA 2016;315:762–74. 26903335 10.1001/jama.2016.0288 PMC5433435

[5] Angus DC van der Poll T Severe sepsis and septic shock. N Engl J Med 2013;369:840–51. 23984731 10.1056/NEJMra1208623

[6] Aird WC The role of the endothelium in severe sepsis and multiple organ dysfunction syndrome. Blood 2003;101:3765–77. 12543869 10.1182/blood-2002-06-1887

[7] Hill JM Zalos G Halcox JP Circulating endothelial progenitor cells, vascular function, and cardiovascular risk. N Engl J Med 2003;348:593–600. 12584367 10.1056/NEJMoa022287

[8] Liao MT Liu WC Lin FH Intradialytic aerobic cycling exercise alleviates inflammation and improves endothelial progenitor cell count and bone density in hemodialysis patients. Medicine (Baltimore) 2016;95:e4134. 27399127 10.1097/MD.0000000000004134 PMC5058856

[9] Rocha NG Sales AR Miranda RL Aerobic exercise modulation of mental stress-induced responses in cultured endothelial progenitor cells from healthy and metabolic syndrome subjects. Life Sci 2015;123:93–9. 25596018 10.1016/j.lfs.2014.12.026

[10] Burtin C Clerckx B Robbeets C Early exercise in critically ill patients enhances short-term functional recovery. Crit Care Med 2009;37:2499–505. 19623052 10.1097/CCM.0b013e3181a38937

[11] Schweickert WD Pohlman MC Pohlman AS Early physical and occupational therapy in mechanically ventilated, critically ill patients: a randomised controlled trial. Lancet 2009;373:1874–82. 19446324 10.1016/S0140-6736(09)60658-9 PMC9906655

[12] Needham DM Truong AD Fan E Technology to enhance physical rehabilitation of critically ill patients. Crit Care Med 2009;37:S436–41. 20046132 10.1097/CCM.0b013e3181b6fa29

[13] Segers J Hermans G Bruyninckx F Feasibility of neuromuscular electrical stimulation in critically ill patients. J Crit Care 2014;29:1082–8. 25108833 10.1016/j.jcrc.2014.06.024

[14] Gerovasili V Stefanidis K Vitzilaios K Electrical muscle stimulation preserves the muscle mass of critically ill patients: a randomized study. Crit Care 2009;13:R161. 19814793 10.1186/cc8123 PMC2784391

[15] Karatzanos E Gerovasili V Zervakis D Electrical muscle stimulation: an effective form of exercise and early mobilization to preserve muscle strength in critically ill patients. Crit Care Res Pract 2012;2012:432752. 22545212 10.1155/2012/432752 PMC3321528

[16] Abu-Khaber HA Abouelela AMZ Abdelkarim EM Effect of electrical muscle stimulation on prevention of ICU acquired muscle weakness and facilitating weaning from mechanical ventilation. Alex J Med 2013;49:309–15.

[17] Rodriguez PO Setten M Maskin LP Muscle weakness in septic patients requiring mechanical ventilation: protective effect of transcutaneous neuromuscular electrical stimulation. J Crit Care 2012;27:319.e1–8. 10.1016/j.jcrc.2011.04.010 21715139

[18] Stefanou C Karatzanos E Mitsiou G Neuromuscular electrical stimulation acutely mobilizes endothelial progenitor cells in critically ill patients with sepsis. Ann Intensive Care 2016;6:21. 26969168 10.1186/s13613-016-0123-y PMC4788669

[19] Poulsen JB Møller K Jensen CV Effect of transcutaneous electrical muscle stimulation on muscle volume in patients with septic shock. Crit Care Med 2011;39:456–61. 21150583 10.1097/CCM.0b013e318205c7bc

[20] Gerovasili V Tripodaki E Karatzanos E Short-term systemic effect of electrical muscle stimulation in critically ill patients. Chest 2009;136:1249–56. 19710290 10.1378/chest.08-2888

[21] Angelopoulos E Karatzanos E Dimopoulos S Acute microcirculatory effects of medium frequency versus high frequency neuromuscular electrical stimulation in critically ill patients: a pilot study. Ann Intensive Care 2013;19:39. 10.1186/2110-5820-3-39 PMC3878255 24355422

[22] Moher D Hopewell S Schulz KF CONSORT 2010 explanation and elaboration: updated guidelines for reporting parallel group randomised trials. Int J Surg 2012;10:28–55. 22036893 10.1016/j.ijsu.2011.10.001

[23] Chan AW Tetzlaff JM Gøtzsche PC SPIRIT 2013 explanation and elaboration: guidance for protocols of clinical trials. BMJ 2013;346:e7586. 23303884 10.1136/bmj.e7586 PMC3541470

[24] Duda DG Cohen KS Scadden DT A protocol for phenotypic detection and enumeration of circulating endothelial cells and circulating progenitor cells in human blood. Nat Protoc 2007;2:805–10. 17446880 10.1038/nprot.2007.111 PMC2686125

[25] Fadini GP Losordo D Dimmeler S Critical reevaluation of endothelial progenitor cell phenotypes for therapeutic and diagnostic use. Circ Res 2012;110:624–37. 22343557 10.1161/CIRCRESAHA.111.243386 PMC3382070

[26] da Rocha EE Alves VG da Fonseca RB Indirect calorimetry: methodology, instruments and clinical application. Curr Opin Clin Nutr Metab Care 2006;9:247–56. 16607124 10.1097/01.mco.0000222107.15548.f5

[27] Clapis FC Auxiliadora-Martins M Japur CC Mechanical ventilation mode (volume × pressure) does not change the variables obtained by indirect calorimetry in critically ill patients. J Crit Care 2010;25:659.e9–16. 10.1016/j.jcrc.2009.11.010 20080021

[28] Lago AF Goncalves EC Silva EC Comparison of energy expenditure and oxygen consumption of spontaneous breathing trial conducted with and without automatic tube compensation. J Clin Med Res 2015;7:700–5. 26251685 10.14740/jocmr2250w PMC4522988

[29] Picolo MF Lago AF Menegueti MG Harris-Benedict equation and resting energy expenditure estimates in critically ill ventilator patients. Am J Crit Care 2016;25:e21–9. 26724304 10.4037/ajcc2016758

[30] Reeves MM Davies PS Bauer J Reducing the time period of steady state does not affect the accuracy of energy expenditure measurements by indirect calorimetry. J Appl Physiol 2004;97:130–4. 15020579 10.1152/japplphysiol.01212.2003

[31] Morete MC Mofatto SC Pereira CA Translation and cultural adaptation of the Brazilian Portuguese version of the Behavioral Pain Scale. Rev Bras Ter Intensiva 2014;26:373–8. 25607266 10.5935/0103-507X.20140057 PMC4304465

[32] Grizzle JE The two-period change-over design an its use in clinical trials. Biometrics 1965;21:467–80. 14338679

[33] Rakocevic J Orlic D Mitrovic-Ajtic O Endothelial cell markers from clinician's perspective. Exp Mol Pathol 2017;102:303–13. 28192087 10.1016/j.yexmp.2017.02.005

